# Fatty Acids Prevent Hypoxia-Inducible Factor-1α Signaling Through Decreased Succinate in Diabetes

**DOI:** 10.1016/j.jacbts.2018.04.005

**Published:** 2018-08-28

**Authors:** Michael S. Dodd, Maria da Luz Sousa Fialho, Claudia N. Montes Aparicio, Matthew Kerr, Kerstin N. Timm, Julian L. Griffin, Joost J.F.P. Luiken, Jan F.C. Glatz, Damian J. Tyler, Lisa C. Heather

**Affiliations:** a Department of Physiology, Anatomy and Genetics, University of Oxford, Oxford, United Kingdom; b Department of Biochemistry and MRC Human Nutrition Research, University of Cambridge, Cambridge, United Kingdom; c Department of Molecular Genetics, Cardiovascular Research Institute Maastricht (CARIM), Maastricht University, Maastricht, the Netherlands

**Keywords:** cardiovascular disease, diabetes, fatty acids, metabolism, HIF-1α, hypoxia, ANOVA, analysis of variance, BSA, bovine serum albumin, DMF, dimethyl fumarate, DMOG, dimethyloxalylglycine, FA, fatty acid, FIH, factor inhibiting hypoxia-inducible factor, HIF, hypoxia-inducible factor, i.p., intraperitoneal, IR, insulin resistance/resistant, MI, myocardial infarction, PHD, prolyl hydroxylase domain, SSO, sulfo-*N*-succinimidyl oleate

## Abstract

Hypoxia-inducible factor (HIF)-1α is essential following a myocardial infarction (MI), and diabetic patients have poorer prognosis post-MI. Could HIF-1α activation be abnormal in the diabetic heart, and could metabolism be causing this? Diabetic hearts had decreased HIF-1α protein following ischemia, and insulin-resistant cardiomyocytes had decreased HIF-1α-mediated signaling and adaptation to hypoxia. This was due to elevated fatty acid (FA) metabolism preventing HIF-1α protein stabilization. FAs exerted their effect by decreasing succinate concentrations, a HIF-1α activator that inhibits the regulatory HIF hydroxylase enzymes. In vivo and in vitro pharmacological HIF hydroxylase inhibition restored HIF-1α accumulation and improved post-ischemic functional recovery in diabetes.

## Highlights

• HIF-1α is activated following myocardial infarction, and is a critical transcription factor promoting survival in hypoxia.
• Type 2 diabetes blunts HIF-1α activation in ischemia and downstream adaptation to hypoxia.
• This effect is mediated by increased long-chain fatty acids, which prevent HIF-1α activation in hypoxia.
• Succinate promotes HIF-1α activation by inhibiting the regulatory HIF hydroxylases. Fatty acids decrease succinate concentrations in hypoxia, by blocking increased glycolysis and malate-aspartate shuttle activity.
• Pharmacologically activating HIF-1α or increasing succinate concentrations restores the hypoxic response and improves functional recovery post-ischemia in diabetes.

## Visual Abstract

**Figure:**
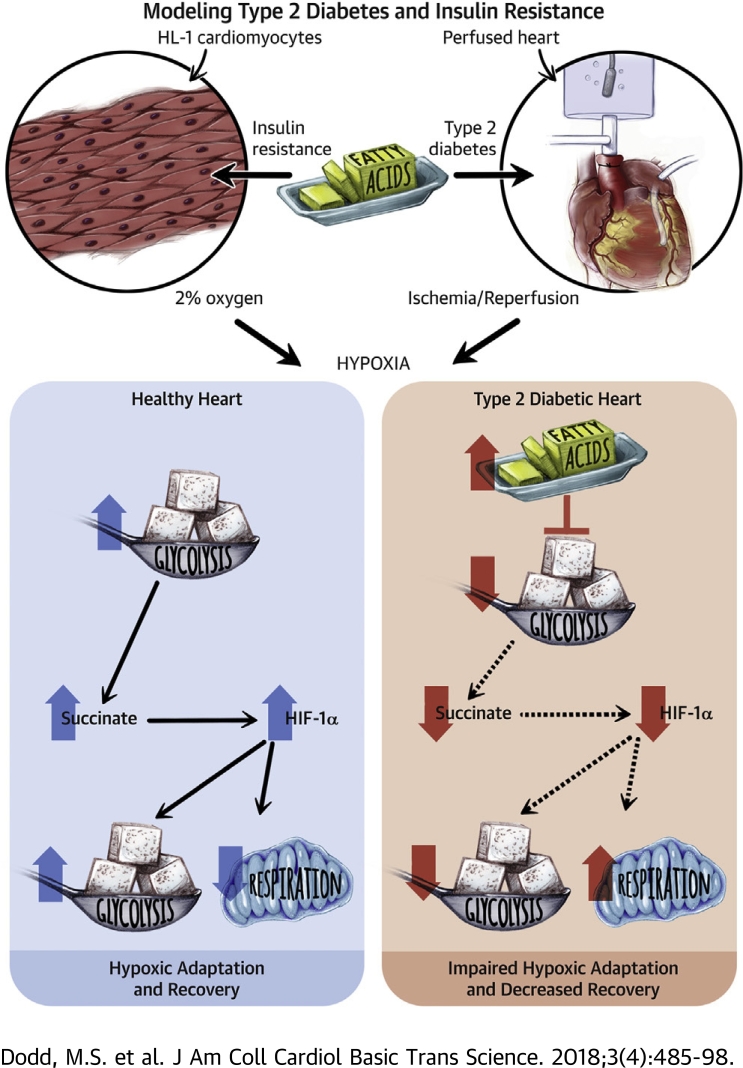

Activation of hypoxic signaling pathways through stabilization of hypoxia-inducible factor (HIF)-1α is vital for cardiac survival in hypoxia, as occurs post-myocardial infarction (MI) or in heart failure 1, 2. In normoxia, HIF-1α protein is hydroxylated by the oxygen-sensing HIF hydroxylase enzymes: prolyl hydroxylase domain (PHD) and factor inhibiting HIF (FIH), thereby targeting HIF-1α for proteasomal degradation (3). During hypoxia, reduced oxygen availability inhibits these HIF hydroxylase enzymes, leading to HIF-1α protein accumulation, which can translocate to the nucleus, bind to HIF1β, and induce transcription of HIF target genes. HIF-1α has many hundreds of gene targets, and broadly, these targets up-regulate oxygen sparing pathways, down-regulate oxygen consuming pathways, and increase oxygen delivery 4, 5, 6, 7. HIF-1α adapts metabolism to the oxygen-restricted environment, increasing anaerobic glycolysis while decreasing fatty acid (FA) oxidation and mitochondrial respiration. HIF-1α plays a critical role in the cellular response to hypoxia in many cardiovascular diseases 1, 2, 8, 9, 10, and decreased HIF-1α activation accelerates the progression into heart failure in genetic mouse models (1).

Cardiovascular disease is the leading cause of mortality in type 2 diabetic patients, with poorer long-term prognosis following MI and higher incidence of heart failure 11, 12, 13. Impaired cardiac function post-ischemia has been associated with abnormal cardiac FA metabolism in diabetes. FAs are the predominant fuel used by the heart to power contraction, accounting for 60% to 70% of cardiac ATP generation. In diabetes, FA oxidation and storage are up-regulated, and lipid intermediates accumulate within the myocardium 14, 15. We have shown that type 2 diabetes blunts the normal metabolic adaptation to hypoxia, resulting in impaired cardiac function (16). However, the explanation for why hypoxic adaption is blunted in diabetes and the underlying mechanisms require investigation, as these may explain why diabetic patients have poorer long-term prognosis following MI and in heart failure. We questioned whether abnormal FA metabolism could drive abnormal HIF-1α activation and downstream adaptation to hypoxia in diabetes.

Here, we show that type 2 diabetic hearts fail to accumulate HIF-1α protein during ischemia, and this is due to elevated FAs. FAs exert their effects by decreasing succinate concentrations, an inhibitor of the HIF hydroxylase enzymes, and supplementing succinate restores HIF-1α accumulation in insulin resistance (IR). Finally, we demonstrate that in vivo HIF hydroxylase inhibition improves post-ischemic recovery in diabetic rats.

## Methods

### Animals

All animal experiments conformed to the Home Office Guidance on the Operation of the Animals (Scientific Procedures) Act, 1986, and were approved by the local ethics committee. Type 2 diabetes was induced as previously described 17, 18, generating a mild model of the disease presenting with hyperglycemia, hyperinsulinemia, and hyperlipidemia (19). Briefly, male Wistar rats were fed a high-fat diet ad libitum for 30 days, and on the 14th day, rats received a single low-dose, intraperitoneal (i.p.) injection of streptozotocin (25 mg ∙ kg^-1^ body weight, w/w in citrate buffer). On day 30, rats were terminally anesthetized using a 0.7-ml i.p. injection of pentobarbital sodium (200 mg/ml Euthatal, Merial Animal Health/Boehringer Ingelheim, Ingelheim, Germany). Blood glucose concentrations was significantly increased from 8.7 ± 0.4 mmol/l in control rats to 10.8 ± 0.5 mmol/l in diabetic rats. Hearts were removed and perfused, according to our published protocol (20), in retrograde Langendorff mode at 100 mm Hg constant pressure. Hearts were perfused with Krebs-Henseleit buffer containing 11 mmol/l glucose, 0.3 U ∙ l^−1^ insulin and 1.5% (w/v) FA-free bovine serum albumin (BSA) bound to 0.4 mmol/l palmitate (gassed with 95% O_2_ and 5% CO_2_, at 37°C). Cardiac function was measured using a polyvinyl chloride balloon inserted into the left ventricle, with rate-pressure product calculated as the multiple of heart rate and developed pressure. Hearts were perfused for 20 min at normal flow, followed by 25 min of low-flow ischemia (0.3 ml/min/gww). At the end of ischemia, hearts were freeze clamped on the cannula for tissue analysis.

For long-term in vivo dimethyloxalylglycine (DMOG) treatment of diabetic rats, on the penultimate week of high-fat feeding, animals were injected i.p. with DMOG (40 mg/kg) every other day over 5 days (totaling 3 injections). After these 5 days of pre-treatment, hearts were perfused for 20 min at normal flow, challenged with 30 min of low-flow ischemia, followed by 15 min of reperfusion, for assessment of post-ischemic contractile recovery.

### Cell culture

HL-1 murine cardiomyocytes were cultured as previously described (21), and all experiments were performed on confluent, beating cells. For induction of IR, cells were washed twice with warm phosphate-buffered saline, before the addition of either control medium (Dulbecco’s Modified Eagles’ Medium) containing 1 g/l glucose, 2 mmol/l glutamine, 2 mmol/l nonessential amino acids, and 1× penicillin/streptomycin) or IR medium (control medium supplemented with 500 μmol/l palmitate:BSA [bound 6:1] with 50 nmol/l insulin) 22, 23. After 8 h, this medium was replenished, and cells were either maintained in normoxia or transferred into a hypoxic incubator (2% O_2_, 5% CO_2_) for a further 16 h. In specific experiments, control medium was supplemented with only FAs (either oleate or palmitate at concentrations from 150 to 500 μmol/l bound to BSA) or with only insulin (50 nmol/l). In other experiments, compounds were added to the media immediately before the cells entered hypoxia for 16 h, which included 1 mmol/l DMOG, 100 μmol/l to 1 mmol/l dimethyl fumarate (DMF), 0.4 mmol/l sulfo-*N*-succinimidyl oleate (SSO), 5 mmol/l 2-deoxyglucose, 2 mmol/l phenylsuccinate, 2 mmol/l amino-oxyacetate, and 5 mmol/l dimethylmalonate. The cell-permeable fumarate ester, dimethyl fumarate, was used to elevate intracellular succinate levels, because this has been shown to be more efficient/cell permeable than the succinate ester, dimethyl succinate (24). For experiments that inhibited HIF degradation, the proteasomal inhibitor MG132 (25 μmol/l) was added, and cells were maintained in hypoxia for 4 h rather than 16 h, due to toxicity effects.

### Western blotting

Cells were washed twice with ice-cold phosphate-buffered saline, before being lysed with the addition of ice-cold lysis buffer. Frozen heart tissue was crushed and lysed in ice-cold lysis buffer. Protein (9 μg for cells and 30 μg for tissue) was loaded onto SDS-PAGE gels and separated by electrophoresis. Even protein loading and transfer were confirmed by either the loading control β-actin or Ponceau S staining. Bands were quantified using LI-COR C-Digit chemiluminescent detection system (LI-COR Biotechnology, Lincoln, Nebraska) and Image Studio software version 5.2.5 (LI-COR).

### Quantitative real-time polymerase chain reaction

Total RNA was extracted from cells using mini-RNeasy kit (Qiagen, Hilden, Germany), and cDNA was synthesized from the RNA using a high-capacity RNA-to-cDNA kit (Thermo Fisher Scientific, Waltham, Massachusetts). Primers were generated by searching PrimerBank for accession numbers of the following genes: *Fih-1* NM_176958.3, *Hif1A* NM_001313919, *Hif1B* NM_001037737.2, *Hprt1* NM_013556.2, *Hmbs* NM_001110251.1, *Phd2* NM_053207.2, *Phd3* NM_028133.2, *Rpl13A* NM_173340.2, and *vhl* NM_009507.3 (PrimerBlast was used to check specificity, and sequences are listed in Supplemental Table 1). *Phd1* is expressed at low levels in the heart, and could not be reliably measured (9). cDNA (15 μg per well) were loaded and quantitative polymerase chain reaction amplification was performed using Power SYBR Green PCR Master Mix and a Step-One Plus Real-Time PCR system (Thermo Fisher Scientific, Waltham, Massachusetts). Relative gene expression was normalized to the geometric mean of the housekeeping genes: *Rpl13a*, *Hmbs*, and *Hrpt1*.

### Respiration measurements

Cells were harvested from flasks with 0.05% trypsin-EDTA at 37°C and counted (using a Countess 1, Thermo Fisher Scientific), and experiments were normalized to viable cell number, expressed per million cells. Respiration was measured using a Clark-type oxygen electrode in medium (100 mmol/l potassium chloride, 50 mmol/l MOPS, 1 mmol/l EGTA, 5 mmol/l potassium phosphate, and 1 mg/ml BSA) (25), supplemented with palmitate (500 μmol/l bound to BSA) and malate (2.5 mmol/l) as substrates. Carbonyl cyanide p-trifluoromethoxyphenylhydrazone (FCCP) (10 mmol/l) was used to measure maximal rates of oxygen consumption, and oligomycin (2 mmol/l) was used to measure ATP synthase-independent proton leak.

### Oil-red-O

Cells were washed and fixed with 4% paraformaldehyde, before being stained with oil-red-O (for neutral lipids) and DAPI (for nuclei) as described (26). Quantification was performed by eluting the oil-red-O in 100% isopropanol and measuring absorbance at 492 nm. To account for cell number, absorbance was normalized to protein content using a bicinchoninic acid assay.

### Media metabolite measurements

An ABX Pentra 400 (Horiba ABX Diagnostics, Irvine, California) was used to measure media glucose and lactate concentrations, and to calculate glucose consumption and lactate efflux rates. Plasma nonesterified fatty acids (NEFA) were measured using a Randox NEFA kit (Randox Laboratories, Crumlin, County Antrim, United Kingdom).

### Metabolomic analysis

Metabolites were extracted from frozen cardiac tissue using methanol–chloroform–water, and the aqueous phase was analyzed as previously described using liquid chromatography tandem-mass spectrometry (19). Glycolytic and Krebs cycle metabolites were semiquantitatively assessed, by normalizing the peak intensity to a number of internal standards. Succinate concentrations were measured in cells using a colorimetric assay (Abcam, Cambridge, United Kingdom), normalized to protein.

### Statistics

**Figure 1:**
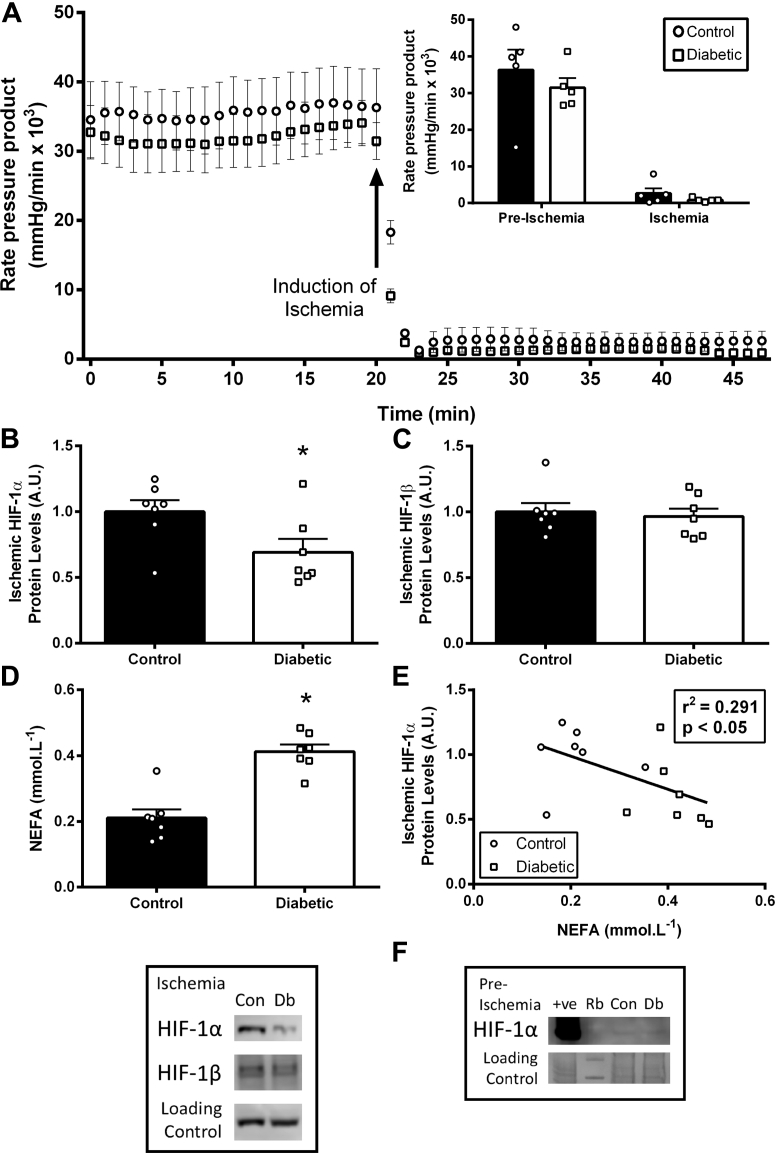
Type 2 Diabetes Decreases HIF-1α Accumulation in Ischemia **(A)** Cardiac function in control and diabetic rat hearts, at normal flow and during ischemia (n = 5 per group). **(B and C)** HIF-1α and HIF-1β protein levels in ischemic control and diabetic hearts. **(D and E)** Non-esterified fatty acid (NEFA) plasma concentrations in control (Con) and diabetic (Db) rats correlated with ischemic HIF-1α protein accumulation. HIF-1α protein in pre-ischemic control and diabetic hearts was minimal, and is shown adjacent to a positive control (hypoxic cells) and rainbow marker (Rb). *p < 0.05 versus control. A.U. = arbitrary units.

**Figure 2:**
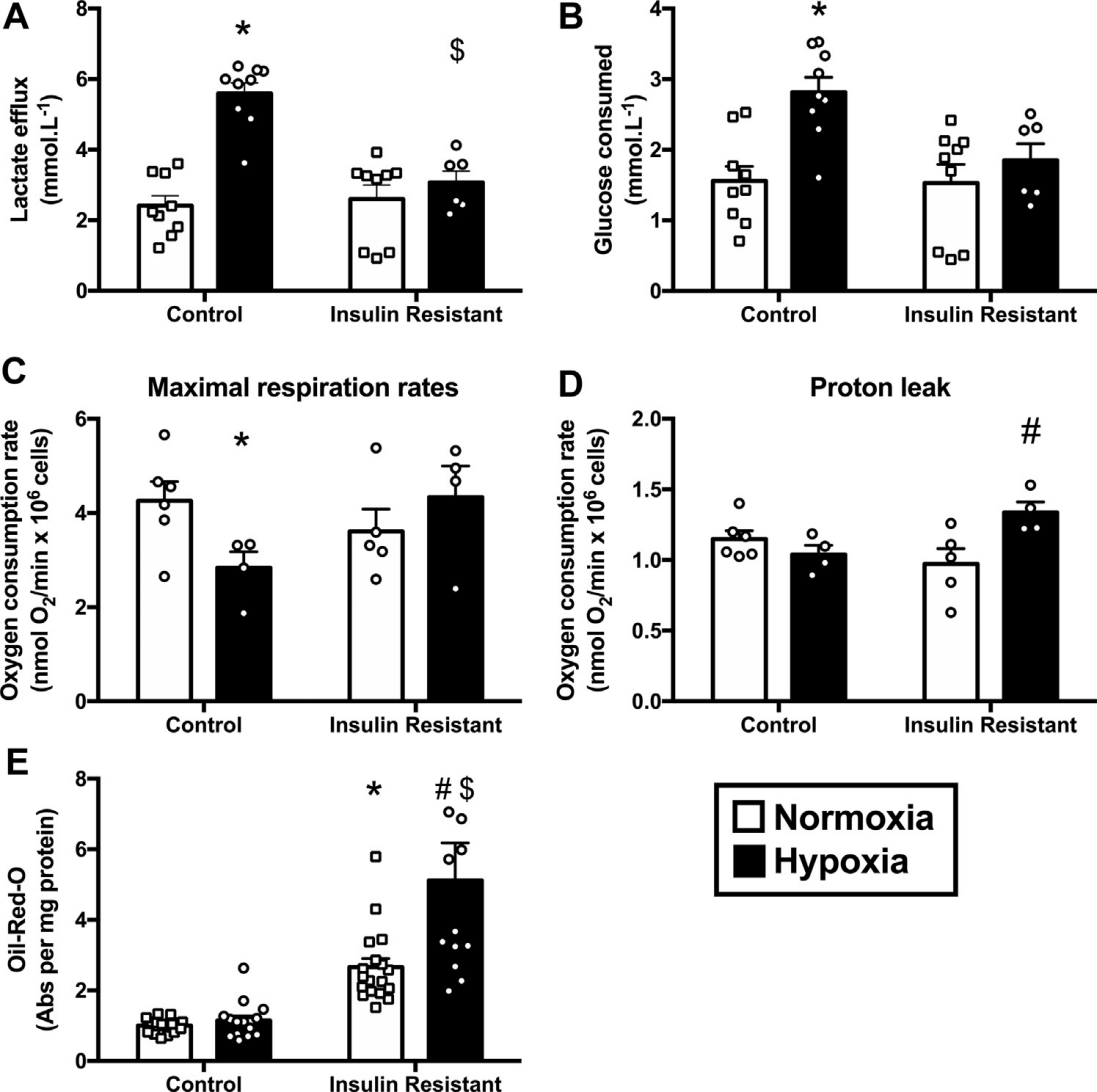
IR Cardiomyocytes Are Unable to Metabolically Adapt to Hypoxia **(A and B)** Lactate release and glucose consumption during normoxia and hypoxia in control and insulin-resistant (IR) HL-1 cardiomyocytes. **(C and D)** Mitochondrial respiration following normoxia and hypoxia in control and insulin resistant cardiomyocytes, measured with FCCP to maximally stimulate respiration or with oligomycin to block ATP synthase activity. **(E)** Staining for neutral lipids using oil-red-O following normoxia and hypoxia in control and IR cardiomyocytes (normalized to normoxic control). *p < 0.05 versus normoxic control, #p < 0.05 versus normoxic insulin resistant, $p < 0.05 versus hypoxic control. Abs = absorbance.

**Figure 3:**
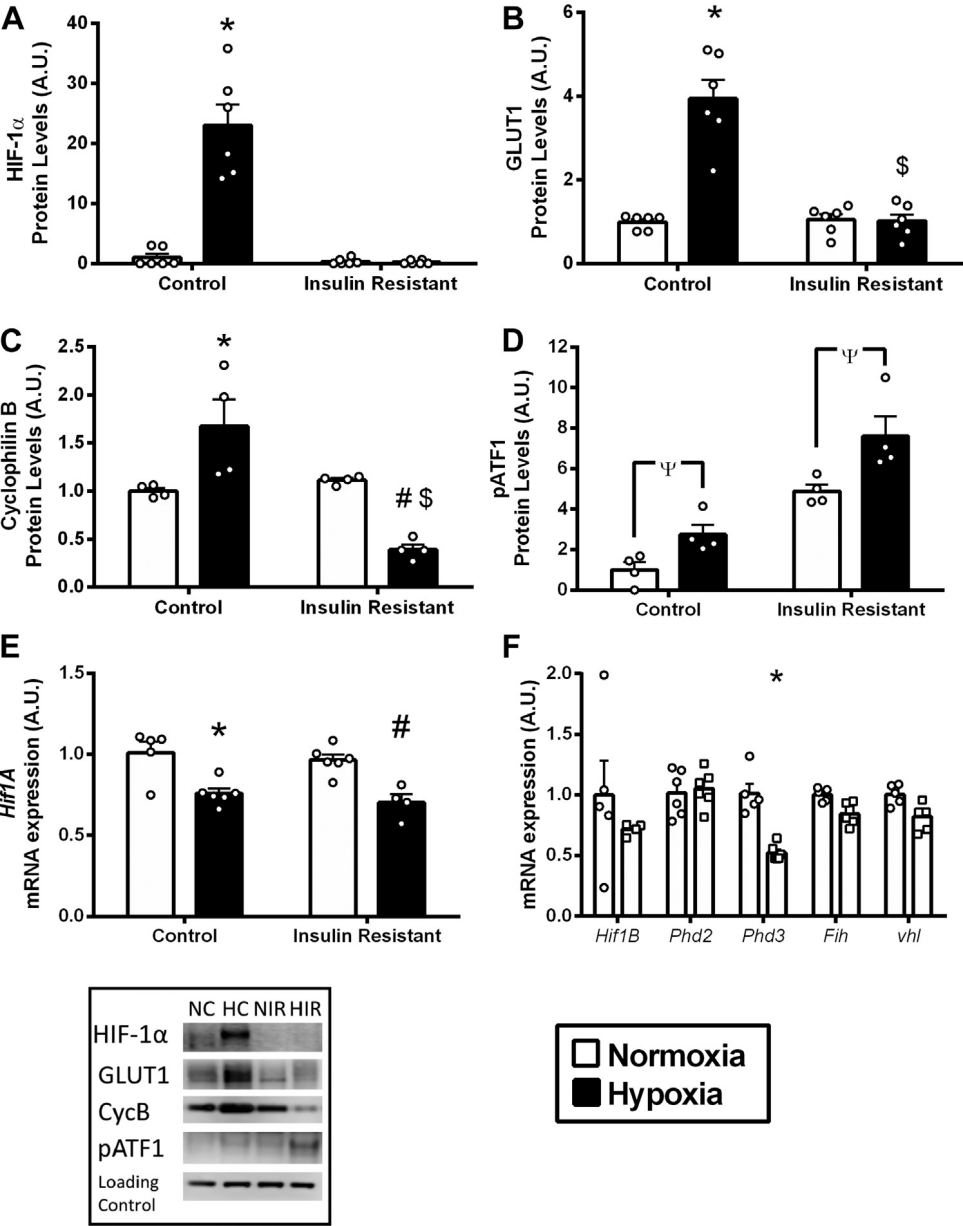
IR Impairs HIF-1α Protein Accumulation and Downstream Signaling **(A to C)** HIF-1α protein accumulation and downstream targets GLUT1 and cyclophilin B protein levels following normoxia and hypoxia, in control and insulin resistant cardiomyocytes. **(D)** Hypoxia-sensitive phospho-activating transcription factor (pATF) 1 protein levels following normoxia and hypoxia, in control and insulin-resistant (IR) cardiomyocytes. **(E and F)** mRNA expression of *Hif1A*, *Hif1B*, prolyl hydroxylase domain enzymes (*Phd*) 2 and 3, factor inhibiting HIF (*Fih*), and von-Hippel Lindau (*vhl*), in normoxic control and insulin resistant cardiomyocytes. *p < 0.05 versus normoxic control, #p < 0.05 versus normoxic insulin resistant, $p < 0.05 versus hypoxic control, Ψp < 0.05 versus effect of oxygen at 2-way analysis of variance. A.U. = arbitrary units.

**Figure 4:**
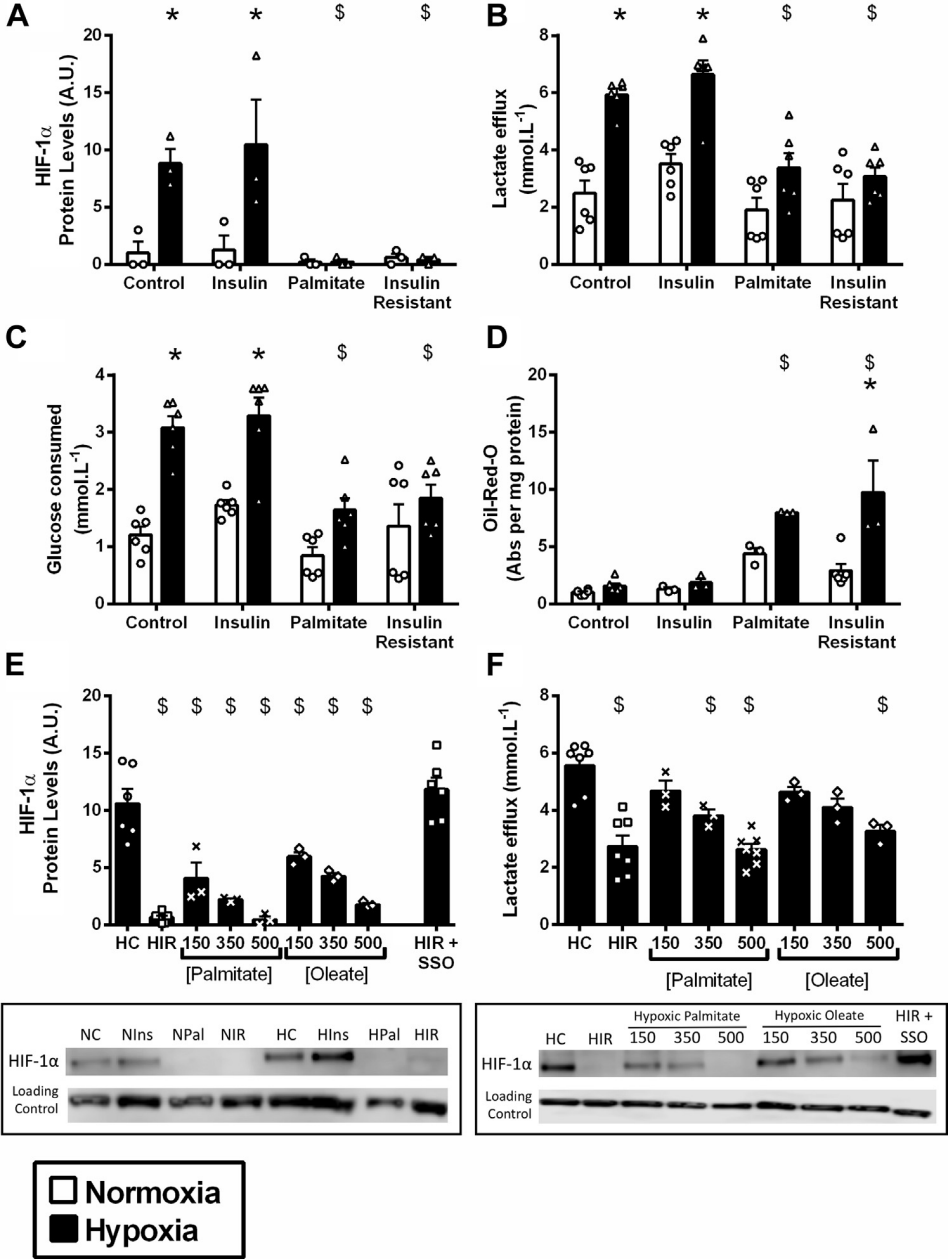
FAs Prevent HIF-1α Accumulation in Hypoxia **(A to D)** HIF-1α protein levels, lactate release, glucose consumption, and neutral lipid oil-red-O staining in normoxia and hypoxia in HL-1 cardiomyocytes cultured in control medium, insulin-resistant medium, and the 2 components that comprise the insulin-resistant medium: high insulin (50 nmol/l) and high palmitate (500 μmol/l). **(E and F)** HIF-1α protein levels and lactate release in hypoxic cells cultured in control medium (HC), insulin-resistant medium (HIR), and control medium supplemented with increasing concentrations of fatty acids (FAs) palmitate or oleate (bound to BSA). **(E)** HIF-1α protein in hypoxic insulin-resistant cells when FA uptake was blocked with sulfo-*N*-succinimidyl oleate (SSO), immediately before the onset of hypoxia (HIR+SSO). *p < 0.05 versus respective normoxic group, $p < 0.05 versus hypoxic control. A.U. = arbitrary units.

**Figure 5:**
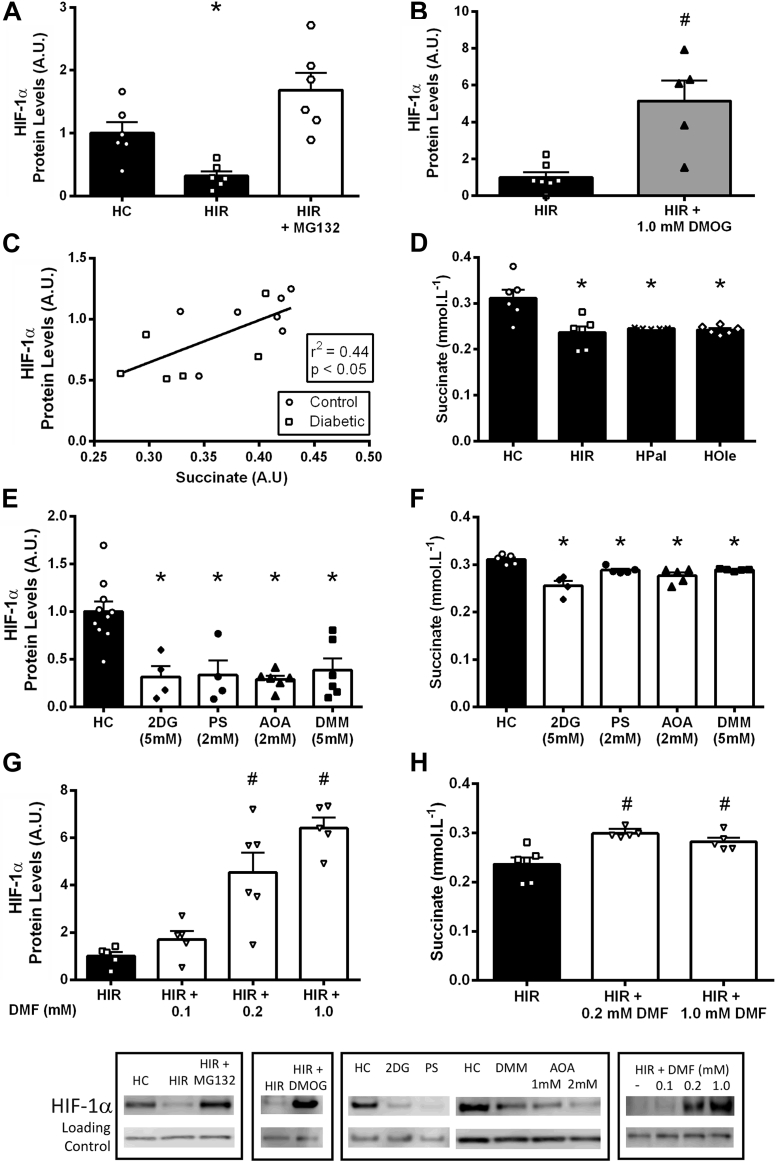
Elevated FAs in IR Decrease Hypoxic Succinate Concentrations Required for HIF-1α Stabilization **(A)** HIF-1α protein levels following acute hypoxia in control (HC) and IR (HIR) HL-1 cardiomyocytes with the proteasome inhibitor MG132. **(B)** HIF-1α protein levels in hypoxic IR cells with or without the HIF hydroxylase inhibitor dimethyloxalylglycine (DMOG). **(C)** In ischemic perfused hearts, HIF-1α protein levels correlate positively with myocardial succinate levels. **(D)** Succinate concentrations following hypoxia in cells cultured in control medium (HC), IR medium (HIR), and control medium supplemented with 500 μmol/l palmitate (HPal) or 500 μmol/l oleate (HOle). **(E and F)** HIF-1α protein levels and succinate concentrations in hypoxic cells in the presence of the glycolysis inhibitor 2-deoxyglucose (2DG), the malate-aspartate shuttle inhibitors phenylsuccinate and amino-oxyacetate (PS and AOA), and the succinate dehydrogenase inhibitor dimethylmalonate (DMM). **(G and H)** HIF-1α protein levels and succinate concentrations in hypoxia in IR cells, with or without the cell permeable succinate donor dimethyl fumarate (DMF). *p < 0.05 versus hypoxic control, #p < 0.05 versus hypoxic insulin resistant. Abbreviations as in Figure 4.

**Figure 6:**
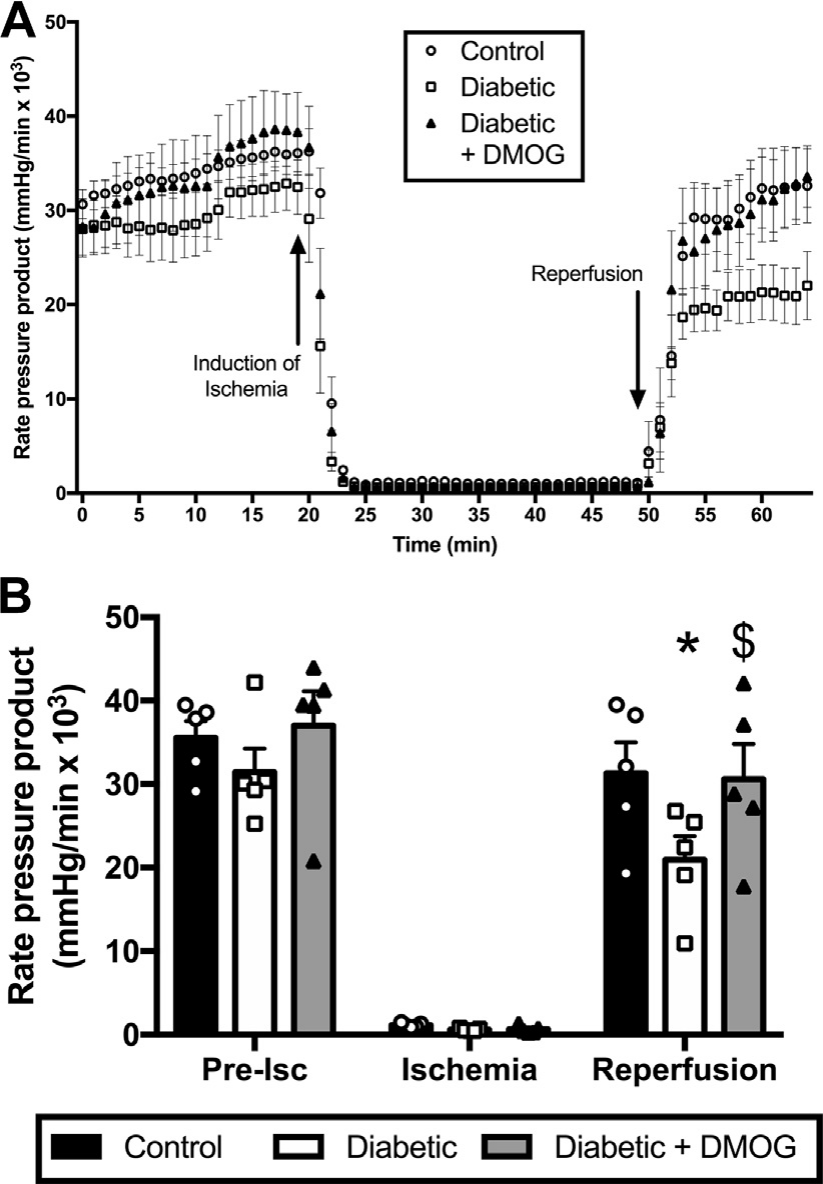
Improved Post-Ischemic Recovery in Type 2 Diabetic Rats Following in Vivo HIF Hydroxylase Inhibition Cardiac function (rate-pressure product) in control, type 2 diabetic and in vivo DMOG-treated type 2 diabetic rats, at normal flow, during ischemia, and following reperfusion (n = 5 per group). *p < 0.05 versus control, $p < 0.05 versus diabetic.

Results are presented as mean ± SEM (Figures 1, 2, 3, 4, 5, and 6). GraphPad Prism version 7 (GraphPad Software, La Jolla, California) was used for statistical tests, and results were considered significant at p < 0.05. Depending on the experiment, either Student’s unpaired *t*-test (Figures 1, 3, and 5), 1-way analysis of variance (ANOVA) (Figures 4, 5, and 6), or 2-way ANOVA (Figures 2, 3, and 4) were performed. Pearson correlations were used for Figures 1 and 5. For the ANOVA analysis, where statistical significances between groups were observed, a Tukey post hoc comparison was performed and corrected for multiple comparisons.

**Figure 7:**
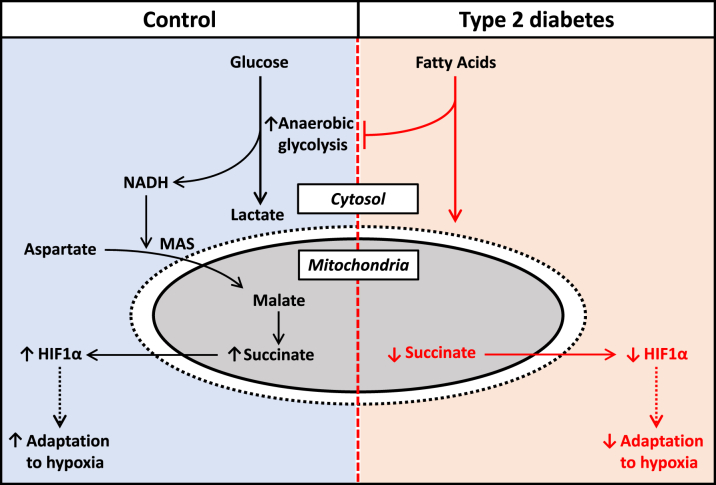
Schematic Detailing the Proposed Mechanism for HIF-1α Suppression in Diabetes In control cells, hypoxia drives increased anaerobic glycolysis, malate-aspartate shuttle (MAS) activity, and reverse Krebs cycle activity. The increased succinate concentrations promote HIF-1α protein stabilization (via inhibition of the HIF hydroxylase enzymes), driving optimal adaptation to hypoxia. In diabetic cells, increased fatty acids in hypoxia inhibits anaerobic glycolysis, resulting in suppressed succinate concentrations and decreased HIF-1α protein stabilization, blunting the adaptation to hypoxia.

## Results

### Type 2 diabetic hearts fail to accumulate HIF-1α during ischemia

Hearts from type 2 diabetic and control rats were subjected to 25 min of low-flow ischemia (Figure 1), and at the end of ischemia accumulation of HIF-1α protein was measured. HIF-1α protein levels were 21% lower in diabetic hearts challenged with ischemia compared with control hearts. The decreased HIF-1α accumulation was independent of any change in HIF1β protein levels. There was a 96% increase in plasma NEFA concentrations in diabetic rats, which correlated negatively with ischemic HIF-1α protein levels. HIF-1α in pre-ischemia/normoxia was similarly absent in both control and diabetic hearts (Figure 1), in agreement with previous findings (16).

### IR cardiomyocytes have blunted metabolic adaptation to hypoxia

To investigate mechanistically why diabetes suppresses cardiac HIF-1α activation, an IR cardiac cell culture model was used 22, 23 (Supplemental Figure 1). Control HL-1 cardiomyocytes exposed to 16 h of hypoxia exhibited the hallmarks of the hypoxic metabolic shift from oxidative to anaerobic metabolism, increasing lactate efflux by 132%, increasing glucose consumption by 81%, and decreasing maximal respiration rates by 33% (Figure 2). By contrast, these hypoxia-mediated metabolic responses were all blunted in IR cells, which failed to increase lactate efflux and glucose consumption. IR cells did not reduce maximal respiration following hypoxia, and instead increased ATP synthase-independent proton leak by 38%. In addition, hypoxia further increased intracellular lipid accumulation in the IR cardiomyocytes.

### IR impairs HIF-1α accumulation and downstream signaling in hypoxia

HIF-1α protein levels were minimal in control and IR cells cultured in normoxia (Figure 3), in agreement with previous findings (16). In control cells, hypoxia caused an ∼23-fold increase in HIF-1α protein levels, causing downstream targets of HIF (GLUT1 and cyclophilin B) to be increased, GLUT1 increased 3-fold, and cyclophilin B increased by 67% 6, 27. By contrast, IR cells failed to accumulate HIF-1α protein in hypoxia, and downstream metabolic HIF targets, GLUT1, and cyclophilin B, did not increase in hypoxic IR cardiomyocytes. Whereas IR cells were unable to activate HIF-1α signaling during hypoxia, there was activation of the hypoxia-sensitive activating transcription factor ATF1 (28). Phosphorylated-ATF1 (pATF1) was elevated by hypoxia, in both the controls and IR cells. This demonstrates that IR selectively impairs hypoxic signaling through HIF-1α protein.

We investigated whether components of the HIF signaling pathway were changed in the IR cells, which would account for the lack of HIF-1α protein stabilization in hypoxia. There was no difference in *Hif1A* mRNA expression between control and IR cells (Figure 3, Supplemental Figure 2). Components of the HIF regulatory pathway did not differ between IR and control cells in normoxia, including HIF-1α heterodimer partner *Hif1B*, the oxygen-sensing enzymes prolyl hydroxylase 2 (*Phd2*) and asparaginyl hydroxylase factor inhibiting HIF (*Fih*), and the von Hippel-Lindau (*vhl*)-E3 ubiquitin ligase that targets HIF-1α protein for proteasomal degradation. *Phd3* mRNA was decreased in IR cells, but this would be predicted to increase HIF-1α protein in hypoxia, not decrease it as observed in the IR cells.

### FAs prevent HIF-1α accumulation in hypoxia in a concentration-dependent manner

IR was induced in our cells by a combination of hyperlipidemia and hyperinsulinemia. The component responsible for impaired HIF-1α activation was investigated by treating cells with either 50 nmol/l insulin or 500 μmol/l palmitate. Hyperinsulinemia alone did not affect HIF-1α activation or the metabolic response to hypoxia (Figure 4). By contrast, hyperlipidemia suppressed HIF-1α accumulation in hypoxia, as exposure to palmitate alone reduced HIF-1α to levels seen in IR cells. In addition, palmitate decreased the downstream HIF-mediated metabolic effects during hypoxia, decreasing lactate efflux, reducing glucose consumption and increasing lipid accumulation in hypoxia. To investigate whether changes were dependent on the concentration or saturation of the FA, cells were incubated with 150, 350, or 500 μmol/l of palmitate or oleate, the 2 most abundant FAs in blood (29). The inhibition of HIF-1α accumulation in hypoxia was proportional to the concentration of FA, and to the same extent whether palmitate or oleate were used. Consistent with the reduced HIF-1α accumulation, there was a failure to increase glycolytic lactate efflux with FA concentrations of 350 μmol/l and above. Finally, we added the sarcolemmal FA uptake inhibitor, SSO, to IR cells immediately prior to hypoxia. Blocking sarcolemmal fat uptake during hypoxia restored HIF-1α accumulation (Figure 4), despite cells remaining IR (Supplemental Figure 1).

### Elevated FAs decrease succinate concentrations, which is required for HIF-1α accumulation

To prevent HIF-1α degradation, we inhibited the proteasome with MG132 in IR cells, and found that proteasome inhibition restored HIF-1α to control hypoxic levels (Figure 5), demonstrating the FA-induced defect was due to increased HIF-1α targeting for degradation during hypoxia. HIF-1α is targeted for degradation by the HIF hydroxylases, which are inhibited by low concentrations of oxygen. Pharmacologically inhibiting these HIF hydroxylases using DMOG during hypoxia significantly increased HIF-1α accumulation in IR cells. Taken together, this demonstrates that in IR, HIF-1α is being incorrectly targeted by the HIF hydroxylases for proteasomal degradation, which should be inhibited in hypoxia.

In cancer cells, in addition to low oxygen, HIF hydroxylases have also been shown to be inhibited by increased succinate concentrations, the product of their hydroxylation reaction 24, 30. Returning to our ischemic hearts, myocardial levels of succinate correlated positively with HIF-1α accumulation (control succinate 0.39 ± 0.02, diabetic succinate 0.33 ± 0.03; p < 0.06) (Figure 5, Supplemental Table 2). In the hypoxic IR cells succinate concentrations were decreased by 24% compared with hypoxic controls, which could be replicated by culturing hypoxic cells with palmitate or oleate.

Succinate could be derived from the malate-aspartate shuttle utilizing glycolytic NADH, coupled to reverse Krebs cycle and succinate dehydrogenase activity (31). To investigate whether this pathway was responsible for regulating HIF-1α stabilization in hypoxia, we pharmacologically inhibited multiple steps in this pathway. In hypoxia, inhibition of glycolysis using 2-deoxyglucose, inhibition of the malate-aspartate shuttle using phenylsuccinate or amino-oxyacetate, or inhibition of succinate dehydrogenase all decreased HIF-1α stabilization to a similar extent. Thus, in hypoxia, succinate is derived from glycolysis driving malate-aspartate shuttle activity. FAs interfere with this process by suppressing glycolysis (Figure 4) and decreasing succinate concentrations (Figure 5). Culturing with the cell-permeable succinate donor, DMF (24), increased succinate concentrations in hypoxic IR cells. In addition, succinate supplementation with DMF increased HIF-1α accumulation in hypoxic IR cells in a concentration-dependent manner, and at 1 mmol/l DMF to the same level as DMOG. Increasing succinate restored HIF-1α accumulation in IR, overriding the inhibitory effects of FAs.

### In vivo HIF hydroxylase inhibition is able to improve post-ischemic recovery in type 2 diabetes

Finally, we questioned whether in vivo HIF hydroxylase inhibition could provide a mechanism to improve post-ischemic recovery in type 2 diabetes. Type 2 diabetic rats were treated in vivo long term with the HIF hydroxylase inhibitor DMOG for 5 days, and after these 5 days, hearts were isolated, perfused, and challenged with ischemia (Figure 6). There were no differences in cardiac function between groups at normal flow or during low-flow ischemia. Untreated diabetic hearts had a 33% decrease in recovery of cardiac function following reperfusion compared with controls. By contrast, treating diabetic rats in vivo with DMOG improved cardiac function by 46% compared with untreated diabetic rats, restoring post-ischemic recovery to levels found in control hearts.

## Discussion

IR impairs cardiac HIF-1α activation during ischemia and downstream adaptation to hypoxia. Here, we demonstrate that this is mediated by FA-induced inhibition of HIF-1α protein accumulation. This is due to increased targeting of HIF-1α for proteasomal degradation by the regulatory HIF hydroxylases. Mechanistically, this is mediated by decreased succinate concentrations from glycolytically driven malate-aspartate shuttle activity, with succinate supplementation restoring HIF-1α signaling in IR. Finally, in vivo inhibition of the HIF hydroxylases restores post-ischemic recovery of cardiac function in diabetic rats.

Decreased HIF-1α protein accumulation was evident in both diabetic hearts challenged with ischemia, and in IR cardiomyocytes exposed to hypoxia. HIF-1α plays a vital role in the survival of cardiomyocytes following ischemia (2), and decreased HIF activation post-ischemia has been shown to accelerate the progression to heart failure in genetic mouse models (1), thus failure to fully activate HIF-1α in diabetic hearts would translate to poorer long-term prognosis post-MI. FAs are the predominant fuel used by the heart to power contraction, accounting for 60% to 70% of cardiac ATP generation. In diabetes, FA oxidation and storage are up-regulated, and lipid intermediates accumulate within the myocardium 14, 15. The IR-induced suppression of HIF-1α accumulation was recapitulated by exposure to FAs. Palmitate and oleate are both long-chain FAs; the former is saturated and often implicated in oxidative damage and apoptosis, whereas the latter is unsaturated and does not induce these deleterious effects (32). Both palmitate and oleate induced the same degree of HIF-1α suppression and in a concentration-dependent manner, demonstrating that this is a conserved response between the 2 most abundant FAs in blood. The relationship between FAs and HIF-1α was further confirmed pharmacologically using the FA uptake inhibitor SSO, which was able to restore HIF-1α accumulation in IR. This study shows for the first time that FAs can regulate HIF-1α stabilization and downstream signaling, and although we demonstrate this effect in type 2 diabetes, this may also have relevance in any condition of increased circulating lipids, for example, cardiovascular disease, familial combined hyperlipidemia, or starvation.

In normoxia, HIF-1α is translated and immediately targeted for proteasomal degradation by a family of HIF hydroxylases, which hydroxylate HIF making it a substrate for destruction by the von Hippel-Lindau ubiquitin-proteasome pathway. These HIF hydroxylases, PHDs and FIH, use oxygen as their substrate, thereby in hypoxia their activity is blunted, and HIF-1α escapes hydroxylation and degradation, instead migrating to the nucleus. IR did not modify HIF-1α mRNA, demonstrating the inhibitory effect of FAs was not mediated via changes in expression of this transcription factor. Instead, HIF-1α protein in IR could be rescued by inhibiting the proteasomal degradation pathway or by additional pharmacological inhibition of the HIF hydroxylases. These data pointed towards the fact that (at 2% oxygen) the HIF hydroxylases are inhibited by more than just low oxygen but also by an additional factor, which was absent in the IR cells.

The HIF hydroxylases are α-ketoglutarate–dependent dioxygenases, which generate CO_2_ and succinate as products of their reaction. In cancer cells, mutations in Krebs cycle enzymes result in accumulation of succinate, which via product inhibition inhibits the HIF hydroxylases, causing HIF-1α accumulation (30). Studies have shown that succinate can inhibit the HIF hydroxylases to promote HIF-1α accumulation, independently of oxygen concentration 24, 30, 33. In healthy hearts, myocardial succinate levels increased 5-fold in response to acute hypoxia (19), and others have shown that in response to ischemia succinate levels increased 4-fold (31). By contrast, we have shown previously that in diabetic hearts, this hypoxia-induced succinate accumulation was blunted (19). In the present study, we demonstrate that this is due to FAs, because culturing cells with palmitate or oleate blunted succinate accumulation. This is further reinforced by findings that pharmacologically suppressing FA uptake in the diabetic heart increases multiple metabolites within the second span of the Krebs cycle (19), and restored HIF-1α accumulation. Replenishing succinate was able to restore HIF-1α accumulation in the presence of FAs, showing that the inhibitory effect of FAs on HIF-1α accumulation could be overcome. This signaling role for succinate, linking metabolism to HIF regulation, occurs during the hypoxic challenge, and provides a mechanism to regulate the transcriptional response when oxygen is chronically restricted. There has been growing interest in the role of succinate, following its recognition as a key driver of reperfusion injury via reverse electron transport when oxygen supply is reinstated (31). These 2 roles for succinate are not mutually exclusive; when oxygen is limited, succinate is playing an adaptive HIF signaling role, but upon sudden restoration of oxygen the succinate is rapidly reoxidized, removing this metabolite signal (19) but with consequent effects on reactive oxygen species generation (31).

At low oxygen, increased glycolysis is coupled to increased malate-aspartate shuttle activity to accommodate the increased cytosolic NADH (31). When oxygen availability is restricted, this mitochondrial malate is diverted backwards around the Krebs cycle resulting in the generation of succinate by reverse succinate dehydrogenase activity (Figure 7) (31). Here, we demonstrate that inhibition of any of these steps—glycolysis, malate-aspartate shuttle activity, or succinate dehydrogenase activity—is able to suppress HIF-1α stabilization in hypoxia. In the present study and previous findings, we have shown that glycolysis and lactate efflux during hypoxia are blunted by diabetes and insulin resistance, facilitated by increased FA metabolism 16, 19, 21. This is mediated by FA-induced Randle cycle inhibition of key targets in glucose metabolism, which can be overcome by blocking FA metabolism 19, 34. This FA-mediated decrease in glycolysis would result in decreased cytosolic NADH (35) and decreased malate-aspartate shuttle activity (36) as reported by others.

Phase 3 clinical trials are currently underway using HIF hydroxylase inhibitors for the treatment of anemia. Here, we demonstrate that this pharmacological approach, using the non-specific HIF hydroxylase inhibitor DMOG, was able to stabilize HIF-1α in IR cells, and improve recovery of cardiac function post-ischemia in diabetic rats. Thus, by promoting HIF stabilization and by overriding the inhibitory effect of FAs on succinate, improvement in cardiac function could be achieved.

## Conclusions

IR prevents cardiac HIF-1α accumulation and downstream hypoxic adaptation in response to hypoxia. This is mediated by elevated FAs in diabetes suppressing succinate accumulation during hypoxia, and increasing succinate concentrations can override this inhibitory effect on HIF-1α stabilization. Pharmacologically inhibiting the HIF hydroxylases promotes HIF-1α accumulation and improves cardiac function following ischemia-reperfusion in diabetic rats.

Perspectives **COMPETENCY IN MEDICAL KNOWLEDGE:** Cardiovascular disease is the leading cause of death in patients with type 2 diabetes, with increased mortality and accelerated progression into heart failure following myocardial infarction. A greater understanding of why this occurs in diabetes and strategies to improve outcome are needed. We report that the lipid-enriched metabolic state of the diabetic heart prevents activation of the master transcription factor hypoxia-inducible factor, and the downstream adaptation to hypoxia needed for cardiomyocytes to survive in an ischemic/hypoxic environment. From a clinical standpoint, impaired activation of hypoxic pathways in diabetes would contribute to decreased recovery following a myocardial infarction.

**TRANSLATIONAL OUTLOOK:** For clinical translation, these observations in cells and animal models should be confirmed at the tissue level in humans with and without diabetes. Due to the exquisite oxygen sensitivity of hypoxia-inducible factor, a challenge for this translation will be how to obtain ischemic human tissue yet avoiding re-oxygenation of the sample. Pharmacologically activating hypoxia-inducible factor following myocardial infarction in diabetes is an exciting prospect from the present data, given that similar compounds are undergoing phase III clinical trials for the treatment of anemia. However, further basic research is required to confirm their repurposing for the diabetic heart.
