# Supplementary material for: Fatty Acids Prevent Hypoxia-Inducible Factor-1α Signaling Through Decreased Succinate in Diabetes
Source: JACC Basic Transl Sci. 2018 Aug 28;3(4):485–98. doi: 10.1016/j.jacbts.2018.04.005 (PMC6115650; doi:10.1016/j.jacbts.2018.04.005)
Supplement: Supplemental Tables 1 and 2 and Supplemental Figures 1 and 2 [file mmc1.pdf]

Supplementary Table 1

Primer List			
Gene	NM	Sense	Anti-sense
Fih-1	NM_176958.3	GTCCCAGCTACGAAGTTACAGC	CAGTGCAGGATACACAAGGTTT
Hif1A	NM_001313919	TGAGCTCACATCTTGATAAAGCTTCT	GGGCTTTCAGATAAAAACAGTCCAT
Hif1B	NM_001037737.2	TTTATCCCTAGAGATGGGTACAGG	CCACAGGCTGGACAGAAACC
Hmbs	NM_001110251.1	TCCCTGAAGGATGTGCCTAC	ACAAGGGTTTTCCCGTTTG
Hprt	NM_013556.2	GACCGGTTCTGTCATGTCG	ACCTGGTTCATCATCACTAATCAC
Phd2	NM_053207.2	AGTCCCATGAAGTGATCAAGTTCA	ATCCGCATGATCTGCATGG
Phd3	NM_028133.2	CAGACCGCAGGAATCCACAT	TTCAGCATCGAAGTACCAGACAGT
Rpl13A	NM_173340.2	CCCTCCACCCTATGACAA	GGTACTTCCACCCGACCTC
vhl	NM_009507.3	CTCAGCCCTACCCGATCTTAC	ACATTGAGGGATGGCACAAAC

Supplementary Table 2

Summary of metabolomics in ischemic hearts								
	Control				Diabetic			
	Average	+/-	S.E.M		Average	+/-	S.E.M	Sig
**Glycolysis**								
Glucose 6 phosphate	0.8807	+/-	0.2399		0.2031	+/-	0.0355	*
Fructose 6 phosphate	0.6969	+/-	0.1748		0.2148	+/-	0.0344	*
Fructose 1,6-bisphosphate	0.1571	+/-	0.0350		0.1118	+/-	0.0188	n.s
Glyceraldehyde 3 phosphate	0.0143	+/-	0.0006		0.0117	+/-	0.0005	*
Dihydroxyacetone phosphate	0.0007	+/-	0.0003		0.0004	+/-	0.0000	n.s
2-phosphoglycerate/ 3-phosphoglycerate	0.6969	+/-	0.1748		0.2148	+/-	0.0344	*
Phosphoenolpyruvate	0.0229	+/-	0.0075		0.0203	+/-	0.0019	n.s
Pyruvate	0.00005	+/-	0.00001		0.00004	+/-	0.00000	n.s
**Krebs Cycle**								
Acetyl-CoA	0.0204	+/-	0.0067		0.0049	+/-	0.0025	Ω
Citrate	0.2510	+/-	0.0577		0.1967	+/-	0.0295	n.s
Aconitate	0.0625	+/-	0.0060		0.0562	+/-	0.0057	n.s
Isocitrate	0.0287	+/-	0.0071		0.0283	+/-	0.0056	n.s
α Ketoglutarate	0.1286	+/-	0.0071		0.1153	+/-	0.0064	n.s
Succinyl-CoA	0.0010	+/-	0.0002		0.0010	+/-	0.0003	n.s
Succinate	0.3914	+/-	0.0289		0.3371	+/-	0.0389	Ω
Fumarate	0.0060	+/-	0.0009		0.0052	+/-	0.0003	n.s
Malate	2.3096	+/-	0.2060		2.1977	+/-	0.1490	n.s
Oxaloacetate	0.010	+/-	0.0034		0.0081	+/-	0.0017	n.s
		n = 6				n = 7		

* $p < 0.05$ vs control, Ω $p < 0.06$ vs control

Data expressed as peak area ratio, relative to internal standard

Supplementary Figure 1

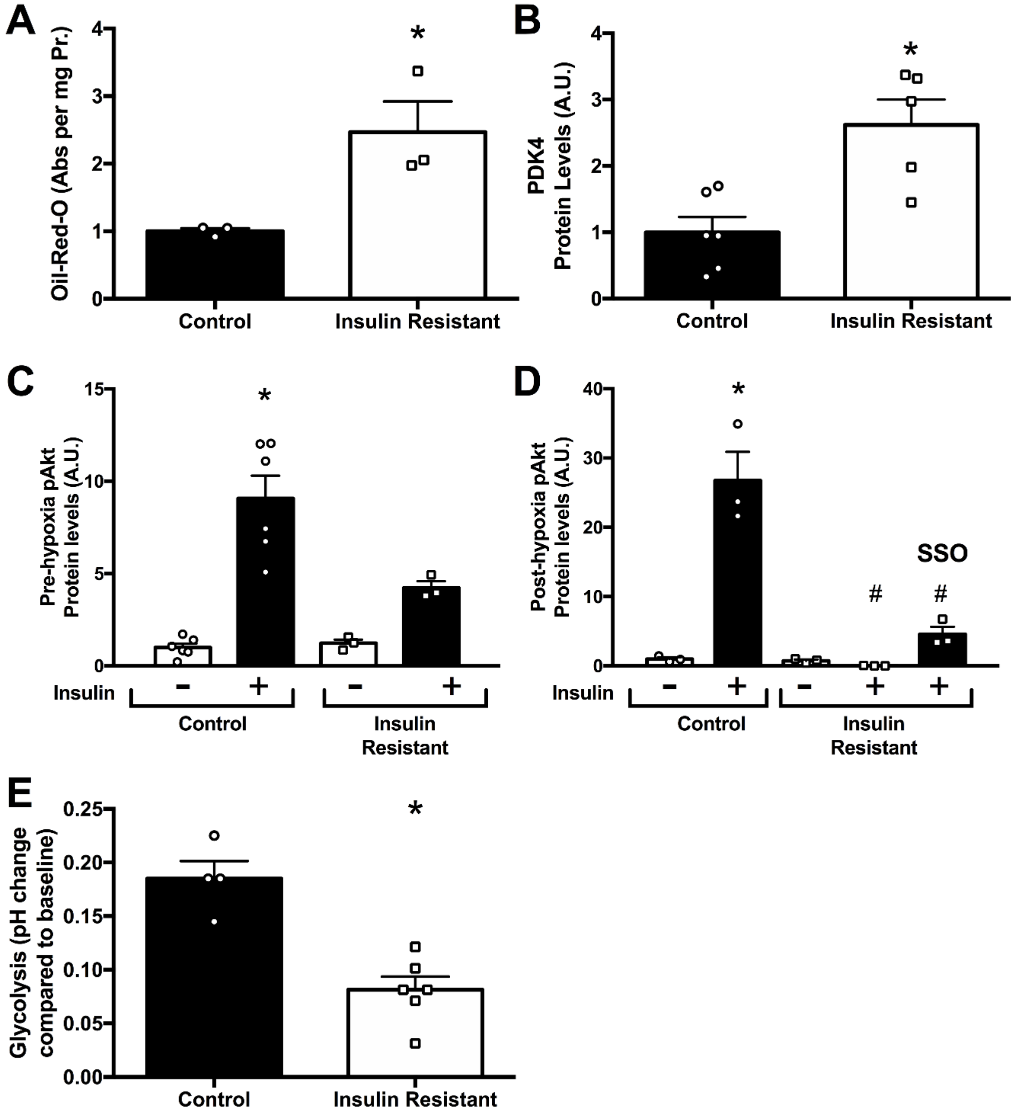

Beating HL-1 cardiomyocytes were cultured with media containing palmitate (500 µM, bound to BSA 6:1) and insulin (50 nM) for 8 hours. Cells had metabolic hallmarks of insulin resistance, including increased oil-red-O staining for intracellular lipids and increased PPARα target protein PDK4 (A and B). In addition, insulin resistant cells had decreased insulin-stimulated glycolytic rates, as assessed using a pH-Xtra glycolysis assay kit (Luxcel Biosciences Ltd) on a fluorescence plate reader FLUOstar Omega series (BMG labtech) analyzed using MARS (BMG labtech) (E). Impaired insulin signaling was demonstrated by decreased insulin-stimulated Akt phosphorylation in response to 30 min of insulin stimulation (200 nM) (C). Insulin resistance was further confirmed after an additional 16 hours in hypoxia by insulin-induced Akt phosphorylation (D). Sulfo-N-succinimidyl oleate (SSO) blocked fatty acid uptake immediately prior to hypoxia, but cells remained insulin resistant as shown by decreased p-Akt in response to insulin stimulation (D). * $p < 0.05$ vs. control unstimulated, # $p < 0.05$ vs. control insulin-stimulated.

Supplementary Figure 2

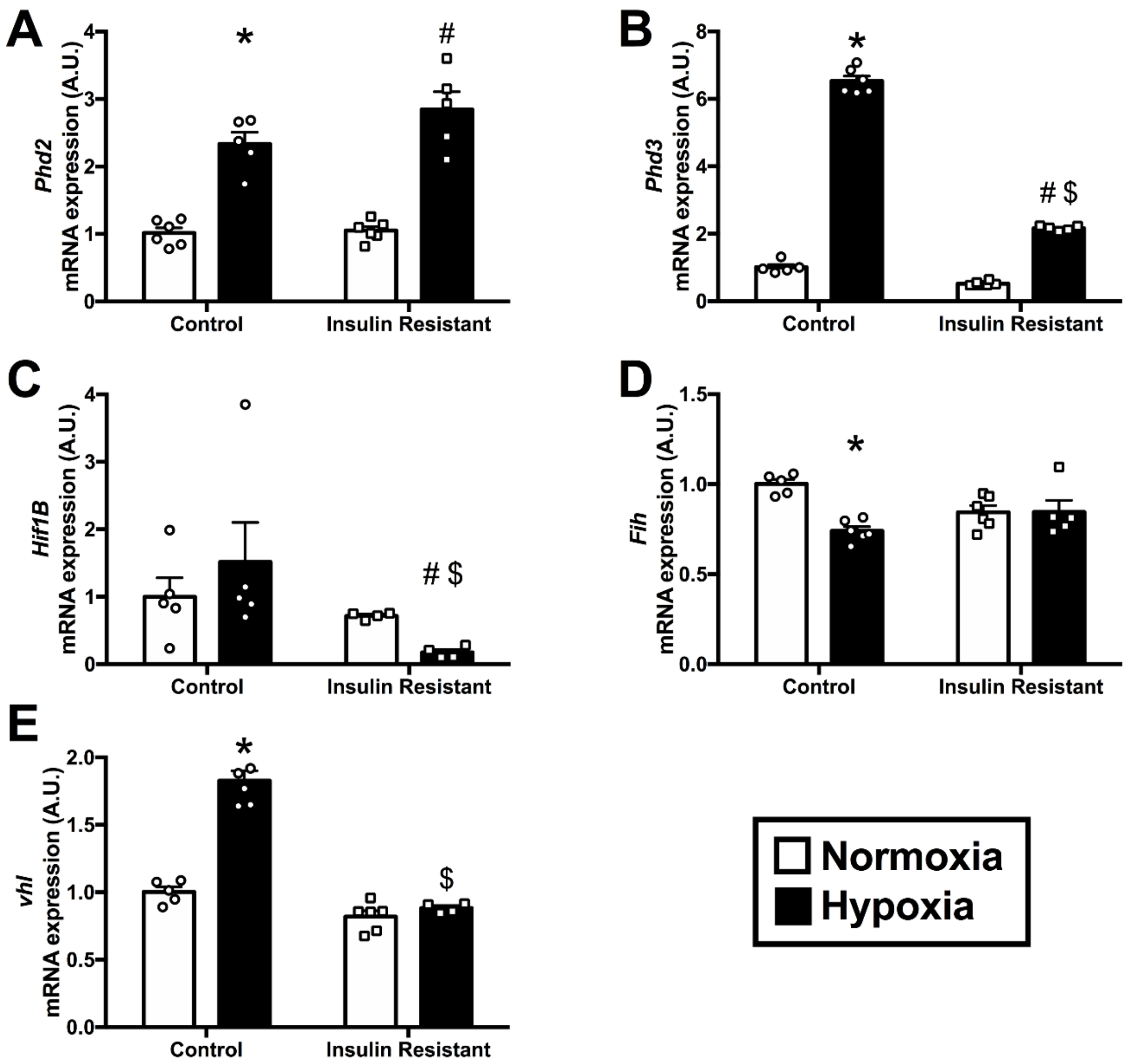

mRNA expression of *Phd2*, *Phd3*, *Hif1B*, *Fih* and *vhl* in control and insulin resistant cardiomyocytes cultured in normoxia and hypoxia (A - E). * $p < 0.05$ vs. normoxic control, # $p < 0.05$ vs. normoxic insulin resistant, $ $p < 0.05$ vs. hypoxic control.
